# Supplementary figures and images for: kMermaid: Ultrafast metagenomic read assignment to protein clusters by hashing of amino acid k-mer frequencies
Source: PLoS Comput Biol. 2025 Sep 11;21(9):e1013470. doi: 10.1371/journal.pcbi.1013470 (PMC12507277; doi:10.1371/journal.pcbi.1013470)

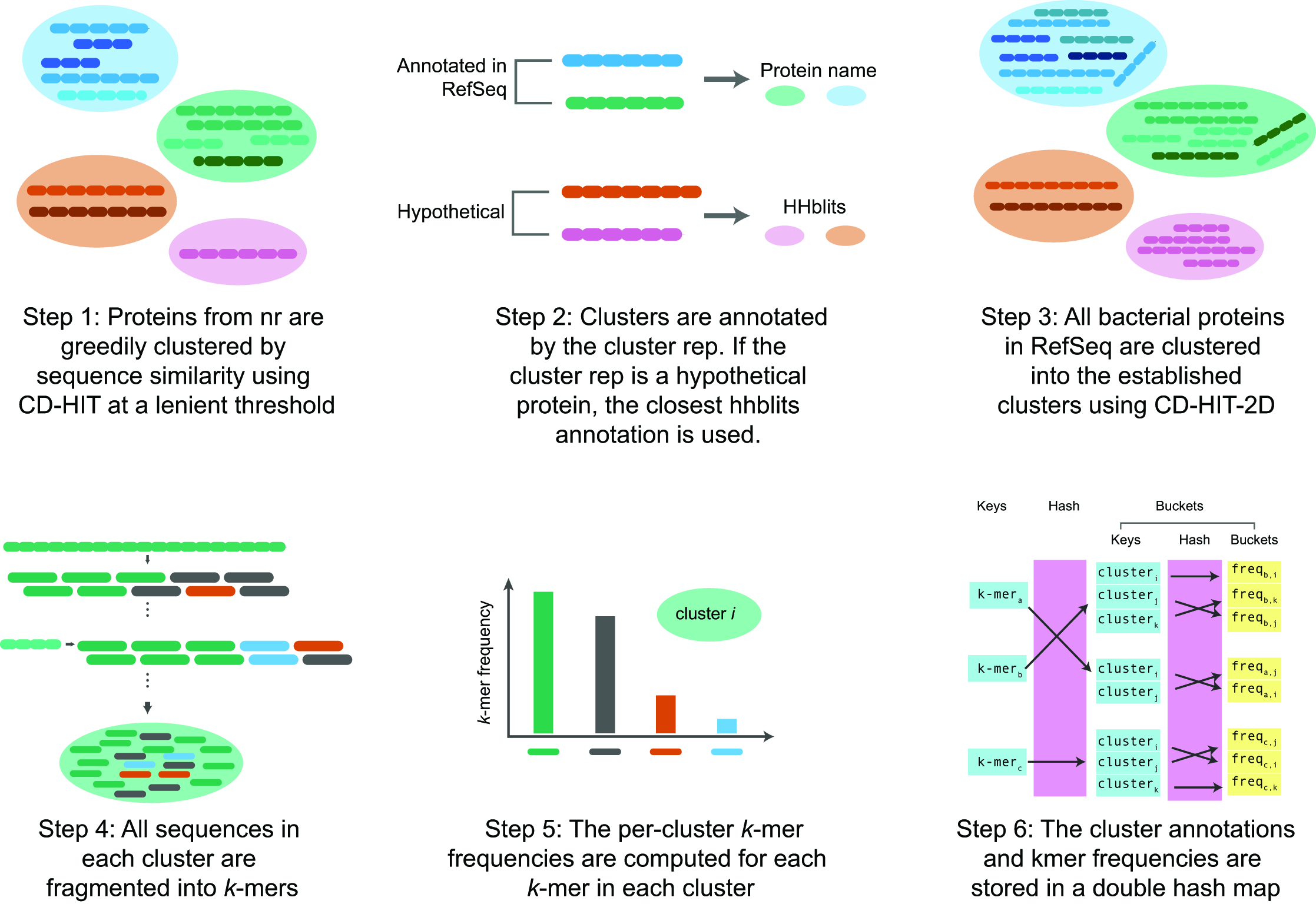

Supplement: S1 Fig — (TIF) [file pcbi.1013470.s001.tif]

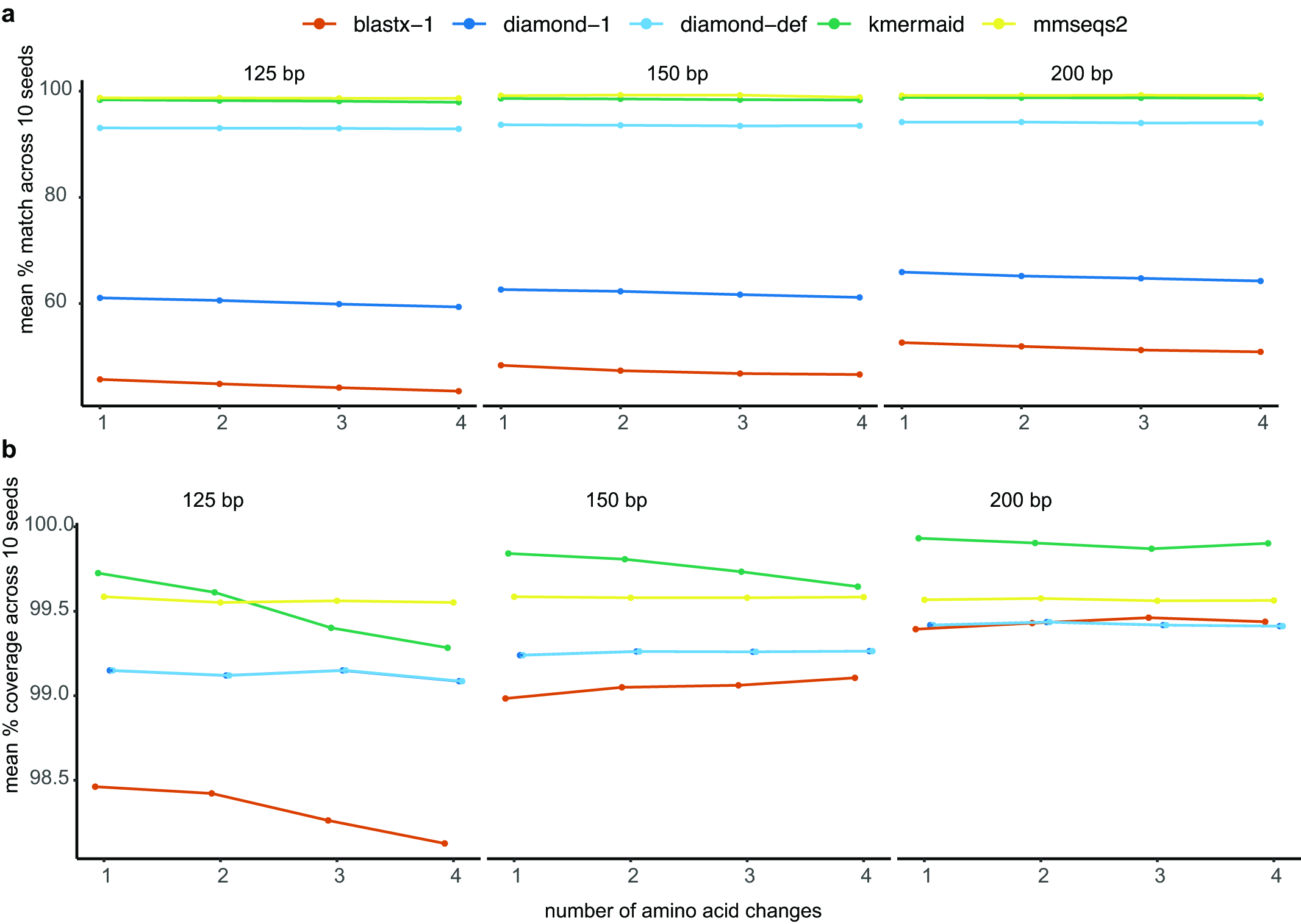

Supplement: S2 Fig — (a) Percent correct labels by each method for 3 typical read lengths evaluated. (b) Percent of input reads mapped by each method for 3 typical read lengths evaluated. (TIF) [file pcbi.1013470.s002.tif]

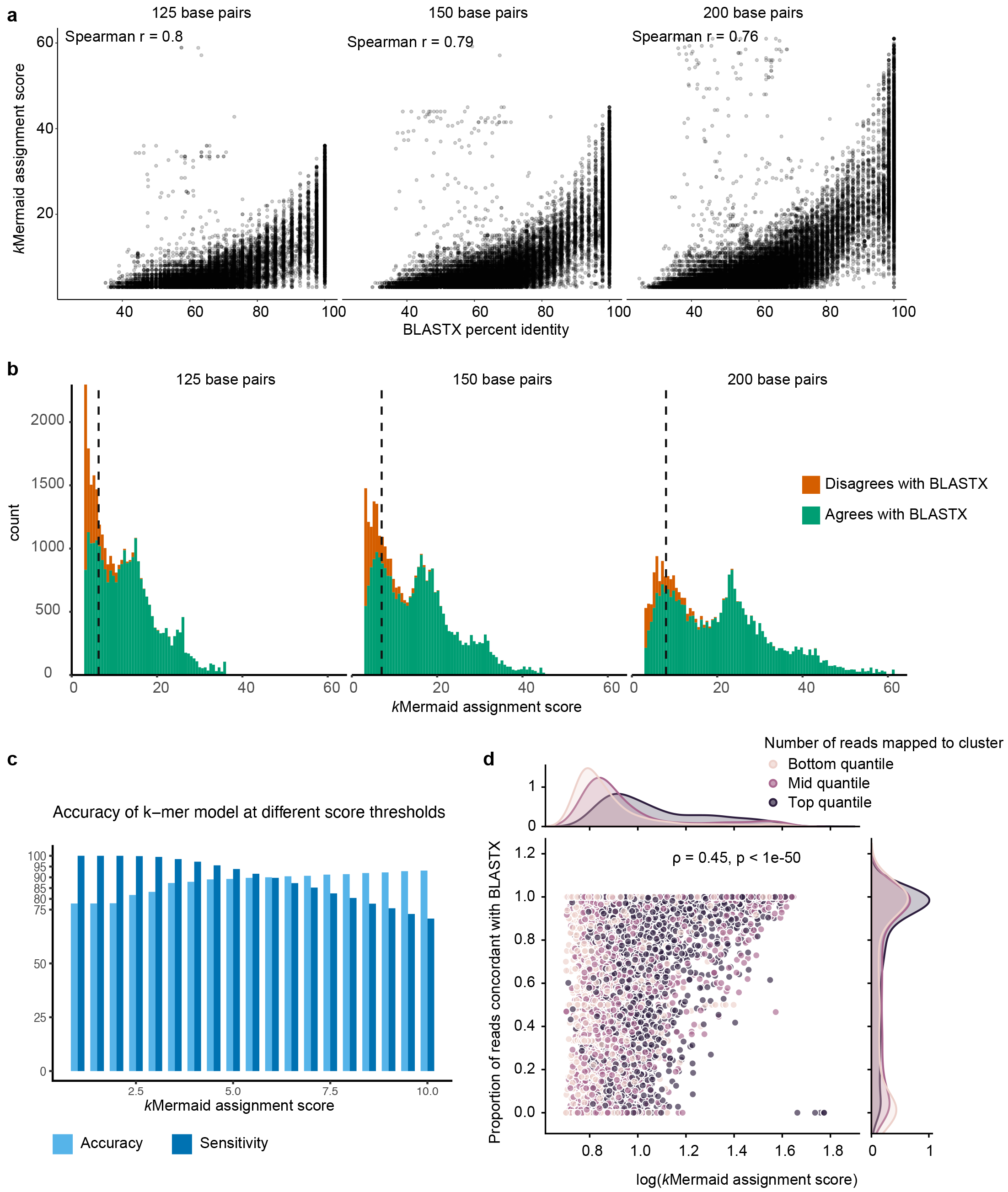

Supplement: S3 Fig — (a) Spearman correlation between kMermaid’s assignment score and the maximum BLASTX percent identity per read for all reverse-translated nucleotide segments (lengths = 125, 150, 200 base pairs) of each unseen RefSeq protein that was mapped by both methods without thresholding. To prevent overplotting, reads were down sampled to 100,000 (40% of total). (b) Histograms showing the kMermaid score of all reverse-translated nucleotide segments (lengths = 125, 150, 200 base pairs) of each unseen RefSeq protein that was mapped by both kMermaid and BLASTX at > 66.6 percent identity. The dashed lines denote the read length-specific thresholds determined by maintaining a false positive rate < 0.05. (c) The percent of all input reads able to be classified by kMermaid compared to BLASTX for sequencing from 3 representative colitis samples, chosen randomly. kMermaid’s optimal scoring threshold was determined by maximizing the percentage of the assignments that agree with BLASTX hits (Sensitivity, dark blue) while retaining a high ratio of assignments that agree with BLASTX to assignments that disagree with BLASTX (Accuracy, light blue). (d) Correlation between the proportion of reads concordant with BLASTX and the mean assignment scores (log-transformed) for all proteins in the cluster. Distributions of these metrics broken down by number of reads mapped to the cluster where clusters in the bottom tertile have the lowest number of mapped reads and clusters in the top tertile contain the highest. (TIF) [file pcbi.1013470.s003.tif]
