# Supplementary material for: kMermaid: Ultrafast metagenomic read assignment to protein clusters by hashing of amino acid k-mer frequencies
Source: PLoS Comput Biol. 2025 Sep 11;21(9):e1013470. doi: 10.1371/journal.pcbi.1013470 (PMC12507277; doi:10.1371/journal.pcbi.1013470)
Supplement: S1 Methods — (PDF) [file pcbi.1013470.s008.pdf]

Supplementary Information: Pseudo-code for model training and read assignment

Algorithm 1 Train k-mer model

```
1: Clusters ← All protein clusters
2: Model ← {w : ∅ | k-mers w}

3: for cluster C ∈ Clusters do
4:     Counts ← {w : 0 | k-mers w}
5:     n_C ← 0
6:     for protein p ∈ C do
7:         n_c ← n_C + 1
8:         for k-mer w ∈ p do
9:             Counts[w] ← Counts[w] + 1
10:        end for
11:    end for

12:    for k-mers w do
13:        if Counts[w] > 0 then
14:            Model[w][C] ← Counts[w]/n_C
15:        end if
16:    end for

17: end for
18: return Model
```

Algorithm 1 describes the process of collecting the frequencies of each k-mer in each cluster, corresponding to Steps 4-6 of Supplementary Figure 1. The result is $Model$, a two-level map. The top-level map stores for each k-mer a map from clusters (containing at least one instance of that *k-mer*) to the frequency of that k-mer in each cluster. $Model$ is initially empty (line 2). $Model$ is implemented using nested Python dictionaries (hash maps); empty entries, corresponding to k-mers that have not been observed in any clusters so far, are not explicitly stored.

For each cluster of proteins C, the method first counts the number of k-mers in each protein in C (lines 4-11). The intermediate $Counts$ (initialized line 4) are also represented as a dictionary and empty entries are not actually stored (so the dictionary is initially empty for each cluster). A list of k-mers in each protein (used in line 8) is pre-computed.

Then, the frequencies of each k-mer observed in C are saved (lines 12-16). Specifically, for each k-mer w, an entry is added to the map for w ($Model[w]$) from C to the number of observations of w in C, normalized by the number of proteins n_C in C (line 14). Because $Counts$ only stores observed k-mers, the iteration and test on lines 12-13 is implemented by simply iterating through entries in $Counts$.

Algorithm 2 Classify a sequence

1: $Clusters \leftarrow$ All protein clusters
2: $Model \leftarrow$ Result of Algorithm
3: $read \leftarrow$ A nucleotide read to classify
4: $score_{best} \leftarrow 0,\ Cluster_{best} \leftarrow$ “”

5: **for** reading frame $frame \in \{+1, +2, +3, -1, -2, -3\}$ **do**
6: $\quad prot \leftarrow$ the translation of $read$ with reading frame $frame$
7: $\quad$ **if** $prot$ is truncated **then**
8: $\quad\quad$ **continue**
9: $\quad$ **end if**

10: $\quad Scores \leftarrow \{C : 0 \mid C \in Clusters\}$
11: $\quad$ **for** k-mer $w \in prot$ **do**
12: $\quad\quad Freqs \leftarrow Model[w]$
13: $\quad\quad$ **for** (cluster C, frequency f) $\in Freqs$ **do**
14: $\quad\quad\quad score_{new} \leftarrow Scores[C] + f$
15: $\quad\quad\quad Scores[C] \leftarrow score_{new}$
16: $\quad\quad\quad$ **if** $score_{new} > score_{best}$ **then**
17: $\quad\quad\quad\quad score_{best} \leftarrow score_{new}$
18: $\quad\quad\quad\quad Cluster_{best} \leftarrow C$
19: $\quad\quad\quad$ **end if**
20: $\quad\quad$ **end for**
21: $\quad$ **end for**

22: **end for**
23: **return** $Cluster_{best}, Score_{best}$

Algorithm 2 describes the process of classifying a nucleotide read (*read*) using k-mer frequencies, corresponding to Figure 1. The result is a maximal cluster $Cluster_{best}$ and corresponding score $score_{best}$, which are tracked on a running basis (initially none and 0, line 4). Each possible translation of *read* into a peptide *prot* is considered (lines 5-6), except that translations which contain stop codons and so are truncated relative to the length of the read are skipped (lines 7-9).

Then, for each protein translation *prot*, the score for each cluster C is computed. These are stored in *Stores* (line 10), a map implemented as a Python dictionary; clusters with score 0 (*i.e.,* those which do not contain any instances of a k-mer yet observed in *prot*, initially all clusters, are represented implicitly and not stored.) The score for a peptide *prot* and cluster C is defined as the sum of the observed frequencies in c of the k-mers in *prot*. As the frequencies of each k-mer w in each cluster are pre-computed in *Model*, this is simply:

$$Scores[C] = \sum_{w \in prot} Model[k][C]$$

To compute scores, the method iterates through each k-mer w in $prot$ (lines 11-21) and retrieves from the top-level map $Model$ a map $Freqs$ of clusters containing w to the frequencies of w in each cluster (line 12). Then, iterating over each cluster C and corresponding frequency (of w) f in $Freqs$ (lines 13-20), the new score $score_{new}$ for C is computed by adding f to the existing score of C, retrieved from $Scores$ (lines 14-15). Then, $score_{new}$ is checked against the best score observed so far ($score_{best}$), and if it is higher, $score_{best}$ is updated along with the corresponding current best cluster $Cluster_{best}$ (lines 16-19).
