# Supplementary material for: kMermaid: Ultrafast metagenomic read assignment to protein clusters by hashing of amino acid k-mer frequencies
Source: PLoS Comput Biol. 2025 Sep 11;21(9):e1013470. doi: 10.1371/journal.pcbi.1013470 (PMC12507277; doi:10.1371/journal.pcbi.1013470)
Supplement: Attachment — Submitted filename: Reviewers-response.docx [file pcbi.1013470.s009.docx]

We thank the reviewers for their insightful comments and suggestions on our manuscript. We have addressed all comments in revision. We believe the revisions have substantially improved this work, further supporting the utility of *k*Mermaid, providing clarification and missing details explaining the method and implementation, and an expanded, comprehensive benchmarking. Please see below a point-by-point response describing how we have revised and improved our manuscript (responses are in red)

Reviewer #1: This work describes a new metagenomic functional profiler tool. The key idea seems to be to first cluster proteins into clusters. However, this is a common approach used by all functional profilers in that they work with existing clusters of orthologous genes (e.g. KEGG OC, EggNOG, COG). Once the clusters are formed, kMermaid seems to use a very standard approach of matching kmers. The approach seems pretty straightforward and perhaps there-in lies its utility.

Specific Comments

1. Why does kMermaid have to rely on its own clusters of proteins? Why cannot it use existing databases?

Thanks for this comment. We generated designated clusters of proteins for *k*Mermaid for two reasons: (1) We have originally investigated the use of existing cluster databases such as PCLA in (Figure 2b) to train our model, but this, as well as other, similar datasets were not as comprehensive as our cluster database. (2) Most importantly, the clustering approach employed in this study is designed to group together proteins with similar AA words. Existing databases do not optimize for this objective. Overall, the approach we employed groups together numerous proteins by AA word similarity, allowing us to use more proteins for the k-mer model while not compromising cluster quality.

We now explain this point in the revised conclusions section:

“Our designated clustering allows assignment of diverse proteins into clusters with homogenous k-mer prevalence, enhancing the potential of the model to correctly capture distinct functions. “

2. The results in figure 2 do not seem very interesting or clear to me. For example, for part a, why would you not use BLASTX results at the cluster level as well? Also part d is hard to interpret. I am not sure I understand what is being conveyed here or that it is important.

We appreciate this comment. This goal of this figure relates to the previous comment of the referee, the point of this figure is to show the utility of the designated clustering employed for *k*Mermaid. Specifically, panel 2a demonstrates that our clusters underlying *k*Mermaid resolves the vast majority of mapping ambiguity (multimappings) observed when using BLASTX (DIAMOND is added in revision), which is a crucial utility of the approach. Indeed, one can assign BLASTX or DIAMOND maps into clusters post-hoc (as done in Fig 2a), which does address the majority of mapping ambiguity but requires additional running time and memory usage for these approaches (whereas *k*Mermaid directly assigns the reads to clusters).

Panels 2b,c demonstrate the functional homogeneity within the *k*Mermaid clusters, i.e. supporting that proteins within the same cluster share similar functions, which is needed as these clusters are established through this work.

Panel 2d demonstrate why these clusters allow sequence assignment by amino acid k-mer similarity, by showing, for example that >50% of kmers are only found in 1 or 2 clusters and thus any read containing such k-mer may be uniquely mapped. We clarify these points in the revised manuscript:

“Out of approximately 2.5M AA 5-mers in our model, presence in a single cluster was the most common scenario (22%) and 81% of all AA 5-mers were found in <10 clusters (Figure 2d). We refer to these 5-mers as “deterministic,” such that presence of a deterministic k-mer in a query sequence is highly informative for cluster assignment. We also found that the AA 5-mers that are present in many clusters have relatively similar frequencies across the clusters when examining the top 10 clusters where they are most frequently found, implying that common 5-mers should not bias the assignment score.”

3. Similarly figure 3 also does not seem to be conveying anything interesting in terms of a result.

Thank you for this comment. We have revised the figures so that currently figure 3 is dedicated to comprehensive benchmarking using simulated data, and figure 4 demonstrate biological applications *k*Mermaid. The revised figure 3 shows a substantially expanded benchmarking analysis, demonstrating the utility of *k*Mermaid compared to different existing approaches with a related objective.

4. Why does the comparison with DIAMOND also not include an evaluation of sensitivity and specificity? Also, why are other recently published tools (e.g. fmh-funprofiler: <https://academic.oup.com/bioinformatics/article/40/Supplement_2/ii165/7749078>) not included in this comparison?

We thank the reviewers for this comment. In revision, we have substantially expanded our benchmarking analysis to include BLASTX, DIAMOND, MMseqs2, which are the most commonly used metagenomic functional assignment methods. Methods were benchmarked for assignment accuracy using real and simulated data, as well as for resource usage.

We agree that fmh-funprofiler is a very useful tool, and we now refer to it in the revised, expanded introduction, in addition to several other useful tools developed for related purposes. However, it is not directly comparable *k*Mermaid because it assigns sequences to KEGG Orthologs, which is a fundamentally different database.

Reviewer #2: This manuscript presents kMermaid, a tool to assign functions to short metagenomic reads. The ideas are interesting and the software is well implemented, but I feel that the authors are overselling the advantages of their approach compared to existing alternatives, mostly by artificially constraining the comparison to only one family of approaches (BLASTX/DIAMOND). The benchmarking is rather simplistic as well.

MAJOR

1. When describing the advantages of kMermaid, the authors claim that it leads to fewer ambiguous mappings, but partly this is achieved by using a different definition of ambiguity so that it applies more strictly when BLASTX is being evaluated than when kMermaid is being evaluated (Fig 2a). For example, if a read is multi-mapped to proteins A1, A2, and A3; but they all share the same cluster, then the read is ambigous at the protein level, but not at the cluster level. This is not novel in the field, but it is should be differentiated what is due to a different tool vs. a different definition of ambiguity.

We thank the reviewer for this important comment. Indeed, a key motivation of *k*Mermaid is to resolve read mapping ambiguity, by providing the user with one-to-one mappings between nucleotide metagenomic sequences and microbial protein annotations. We agree that the notion of using clusters rather than individual proteins is not novel, however, use of inter-cluster k-mer homology model for ultrafast assignment of each read into a unique cluster is novel. We revise the text throughout the manuscript to clarify this point, for example:

“The performance of methods that align to a single protein increase substantially when viewing the singly aligned results at the cluster level in concordance with the notion that clustering proteins by homology resolves ambiguous alignments in most cases.”

“We show that using a well-known concept of clustering homologous proteins into a single functional unit, *k*Mermaid rapidly resolves the majority of ambiguous BLASTX proteins alignments while retaining the granular functional information”

“To circumvent this issue, *k*Mermaid uses a well-defined concept and groups functionally related proteins into functional units prior to assignment such that a read can be uniquely classified into a single functional unit”

BLASTX is designed to be very sensitive as well (compared to other alternatives, such as DIAMOND or mapping to nucleotide databases, such as gene catalogs), thus it will map reads to more proteins.

We agree with this point in principle. However, in practice, we used a very stringent threshold in the number of maps allowed by BLASTX, which is comparable to DIAMOND results. We demonstrate this in the revised figure 2a, by additionally comparing the number of hits to that of DIAMOND vs. *k*Mermaid clusters. As shown, the proportion of reads that are multi-mapped is similar between DIAMOND as BLASTX using these stringent parameters.

2. I disagree that "BLASTX remains the gold standard" for querying metagenomic reads against a large database. Despite having published many papers using metagenomics data, I have never used BLASTX for this purpose. If working in a reference-based manner, I would use nucleotide-alignment (such as bwa or strobealign) to a gene catalog (either the venerable IGC if working with the human gut [<https://doi.org/10.1038/nbt.2942>] or a more recent one [<https://doi.org/10.1038/s41586-021-04233-4>]) or a catalog of genomes (including MAGs, [<https://doi.org/10.1038/s41587-020-0603-3>]). Alternatively, the authors could use the set of 1,793,361 sequences they considered (after dereplicating at 97% or 95% nucleotide identity). I would use eggnogmapper [<https://doi.org/10.1093/molbev/msab293>] to assign functions to the genes. At the very least, the authors need to acknowledge that there are other approaches that are widely used in the field and, ideally, compare their tool to these alternatives.

We thank the reviewer for this comment, and we agree with this point. In revision, we revised this sentence, and we substantially expanded the introduction to discuss newer, related functional mapping tools including fmh-funprofiler, KOALA methods, eggnogmapper and others. We also clarified the distinction between *k*Mermaid and functional assignment tools for related albeit different purposes (such as pathway assignment, etc.). The revised section reads:

“Functional read classification is a broad notion that can encompass both mapping reads directly to proteins or mapping to higher level functional classes, such as ortholog groups or pathways. There are several newer methods and pipelines, such as eggNOG-mapper^6^, PANNZER2^7^, BlastKOALA^8^, HUMAnN3^9(p3)^, and fmh-funprofiler^10^, which have been developed to perform the latter higher-level classifications. Such tools use a variety of methods, such as alignment and *k*-mer sketching, and report mappings to functional reference databases, such as KEGG Orthology or eggNOG^11^. Alternatively, direct protein mapping is often performed using alignment-based methods, which rely on homology between a metagenomic sequence and microbial proteins in reference databases^5^ BLASTX^12^ remains the gold standard for sensitivity despite it being infeasibly slow for typical metagenomic experiments producing tens of millions of reads^13^. DIAMOND^14,15^ was developed in part to address computational challenges associated with BLASTX, allow for ultrafast and read-to-protein alignments, making it one of the most widely used metagenomic protein mapping tools. Another popular method, MMseqs2^16^, was primarily developed to cluster metagenomic nucleotide and protein sequences, but also has protein search capabilities. While ultra-fast, neither DIAMOND nor MMseqs2 protein mapping address BLASTX’s other challenge of multimapping, i.e. when a single read aligns to more than one protein.”

Furthermore, we substantially expanded the benchmarking comparisons to include newer functional assignment methods that are comparable to kMermaid. eggnogmapper was not included the benchmarking because it is not recommended for unassembled read classification, and it is using DIAMOND in BLASTX mode or MMseq2 internally for protein assignment, which is already represented as a standalone comparison.

3. The authors use the term "functional", but they are simply assigning to a protein cluster at 65%/70% identity. Why is this a good proxy for function? Normally, when discussing function, an orthologous group is used (KEGG being the most popular, but also eggNOG, Pfam, ... or function-specific databases such as CARD for antibiotic resistance, CAZy for carbohydrate enzymes, ...). The authors should clarify this point.

We thank the reviewer for this comment, and we agree with the point that “functional” could be too nebulous of a term. In revision, we changed the wording across the manuscript from “functional” clusters to “homologous protein clusters” and “protein (/cluster) mapping” instead of “functional mapping” as well as clarified that the clusters are homologous in protein sequences. We additionally added a brief discussion of different frameworks for functional mapping more generally. We believe the text now more precisely reflects the purpose of *k*Mermaid and its distinction from other functional mapping tools.

4. A typical test that is missing is to remove certain taxonomic groups from the database while using them in simulation (to mimic the case where the real world contains species/strains absent in the database). While still limited, this would be closer to a real-world scenario. I generally refrain from asking for specific experiments in reviews, but this paper is really lacking in having a more realistic benchmark.

We appreciate this suggestion. We agreed that such testing on proteins not included in training is important. However, all proteins in the database were used to define the clusters, which is a time limiting step. Therefore, to simulate a real-world scenario, we provide an additional benchmarking analysis using reads from proteins that were added to RefSeq in 2025, after the completion of the cluster model, and were thus absent from the database. The results of this analysis are shown in revised Figure 4a.

MINOR

"human samples" -> "human gut samples"

Thank you, this has been corrected.

Reviewer #3: The manuscript introduces kmermaid, which is capable of performing functional classification of metagenomes. It focuses on speed, low memory consumption and disambiguation when assigning metagenomic reads against a pre-calculated database of protein clusters.
It avoids the effort of taxonomic disambiguation by performing clustering of reads translated to their amino acid sequences and counting k-mer matches against precomputed protein clusters. The algorithm is rather simple: a single read (not assembled) is translated into all possible reading frames, and the contained k-mers are compared to a set of precomputed protein clusters in what looks like a rather brute-force approach (no indexing/hashing/sorting is mentioned). The cluster with most exact matches is chosen for functional annotation transfer.

The paper is well written and the code it is accompanied with is well structured and organized. However there are a number of major concerns I have with the manuscript.

MMseq2 [1], DIAMOND, Super-FOCUS and FMAP (Fast Metagenomic Functional Profiling) also address the same research question and are established methods (several years old). The authors should carve out their novelty with respect to them and/or ideally quantify performance measures such as speed and accuracy (the current version only contains runtime comparison to DIAMOND).

The introduction does not comprehensively summarize the state of the art (except for DIAMOND). The benchmark should be designed such that a more direct and comprehensive comparison is possible. Ideally, the same benchmarks from e.g. MMseq2 could be used. Thus the benchmarking with the state of the art is not rigorous/comprehensive enough.

Thank you for this comment. In revision, we have substantially expanded the introduction to discuss recent approaches, and better explain the difference between *k*Mermaid and existing functional mapping tools. As suggested, we also substantially expanded the benchmarking comparisons, by including DIAMOND and MMseqs2 across comparisons (now shown in revised Figure 3). Furthermore, we provide an additional benchmarking analysis using reads from proteins that were added to RefSeq in 2025, and where thus not included in our database and cluster model (provided in revised Figure 4a). The revised introduction section is copied below:

“Functional read classification is a broad notion that can encompass both mapping reads directly to proteins or mapping to higher level functional classes, such as ortholog groups or pathways. There are several newer methods and pipelines, such as eggNOG-mapper^6^, PANNZER2^7^, BlastKOALA^8^, HUMAnN3^9(p3)^, and fmh-funprofiler^10^, which have been developed to perform the latter higher-level classifications. Such tools use a variety of methods, such as alignment and *k*-mer sketching, and report mappings to functional reference databases, such as KEGG Orthology or eggNOG^11^. Alternatively, direct protein mapping is often performed using alignment-based methods, which rely on homology between a metagenomic sequence and microbial proteins in reference databases^5^ BLASTX^12^ remains the gold standard for sensitivity despite it being infeasibly slow for typical metagenomic experiments producing tens of millions of reads^13^. DIAMOND^14,15^ was developed in part to address computational challenges associated with BLASTX, allow for ultrafast and read-to-protein alignments, making it one of the most widely used metagenomic protein mapping tools. Another popular method, MMseqs2^16^, was primarily developed to cluster metagenomic nucleotide and protein sequences, but also has protein search capabilities. While ultra-fast, neither DIAMOND nor MMseqs2 protein mapping address BLASTX’s other challenge of multimapping, i.e. when a single read aligns to more than one protein.”

The main algorithm isn’t presented formally and Figure 1 lacks detail and rigor. Subfigures with labels a-e (like in some of the other figures) would be desirable, also for a more structured caption of Figure 1. Alternatively, pseudo-code could be accompanied. It is not entirely clear what is meant with ‘truncated reads’ (together with the X-shaped symbols) – it could be truncation due to instrumental limitations from sequencing technology, low quality bases, incomplete synthesis (premature termination) during sequencing or the presence of a stop codon – which one is it?

We appreciate this comment and agree that the method description lacked detail and required considerable improvements. We have revised Figure 1 to include substantially more detail, such as clarifying which frames are being discarded. We also include a corresponding supplemental figure 1, detailing the underlying cluster model. Furthermore, we provide supplementary material with a complete pseudo-code for training the k-mer model and classifying a sequence using the model. In addition, we provided more detail to the relevant Methods sections. Overall, in the revised manuscript we provide in-depth description of the clustering and read assignment procedures and our specific implementation, better supporting the utility of the approach and providing reasoning for the demonstrated improvement in running time.

Figure 2 claims that BLASTX has a large amount of ambiguous functional maps. 1. It is not clear from the manuscript whether the BLASTX ambiguity is derived from raw scores or E-values (or something else altogether), as it seems that raw scores would also lead to less ambiguous maps. 2. How about the other state of the art methods? Do they also exhibit such high levels of ambiguity?

In revision, we clarified the parameters selected for BLASTX searches presented Figure 2a in the revised Methods section. This revised methods section is copied below:

“For consistency, we used the default or recommended configurations of each method. BLASTX and DIAMOND in BLASTX mode were run with an e-values of 1e-4 and 1e-3 (default), respectively. Mmseqs2 was run with min-length set to 16 and e set to 1e-4. Each method except for BLASTX was set to report the default number of matches based on the e-value. BLASTX was set to report maximum 5 matches due to its excessive running time when reporting all matches. BLASTX and DIAMOND were additionally run with maximum match of 1 to demonstrate the consistency of mapping when using higher level groups rather than exact matches as well as to provide a fairer comparison resource benchmarking.”

We have also clarified in revision that the multimaps are primarily consisting of the same protein originating in multiple different taxa:

“Multimapping is often due to homologous proteins originating in different taxa, but can be problematic for downstream read counting and subsequent analysis^17–19^“

Furthermore, in revision we included DIAMOND in this comparison, which results in similar trend, presented in the revised Figure 2a.

Fig 2a) how are the 3 metagenomic samples chosen?

In the previous version of the manuscript, we chose those 3 samples by random, and these were the only samples for which we could complete the running of BLASTX. In revision, to provide a more complete comparison, we replaced this analysis using all samples from this SRA project, employing random down-sampling to allow computational feasibility. These results are highly consistent across samples (revised Figure 4b), as well as with the results from the initial 3 samples. We retained original 3 samples with the full BLASTX results as a “sub-cohort” for more comprehensive analysis and moved these analyses to the Supplementary (revised Supplemental Figure 3c).

2c) It seems odd that the x-axis exceeds 100% (probably an artefact from KDE)

Indeed, this is an artifact of the KDE plot because the mean of the distribution is so close to 100%.

2d) the x-axis has k-mers “sorted”. It would be best to make clear that the sorting isbased on the number of clusters, a k-mer appears in. When the text refers to Figure 2d) it mentions a single cluster being common (22%), this is not obvious from the Figure, but it could be marked. Likewise the 19% of k-mers falling into >10 clusters

Thank you for this comment. In the revised manuscript we clarified how the k-mers are sorted in the legend of revised Figure 2d.

How did the authors come to 2.5M k-mers. There are approx. 205 =3.2 possibilities for 5-mers. In general the choice of k is not fully motivated

Thank you for this comment. We have clarified that the 2.5M k-mers are 5-mers naturally occurring in proteins in our underlying cluster model rather than all possible 5-mers:

“This precomputed model, consisting of 2,574,615 unique 5-mers which are all the naturally occurring AA 5-mers in the underlying protein cluster database, is then used to determine the cluster to which a query sequence is assigned.”

In addition, in revision we describe the hyperparameter search analysis which led to the selection of the k value:

“The value of k was chosen by using a hyperparameter search, by simulating truncations of each protein in the database into 50AA overlapping segments. k values of 3,4,5,6, were examined, and k=5 was selected as it optimized the performance on truncated reference AA sequences.”

The method section does not motivate some of the design choices made:
• Why 65% (1. Step) and 70% (2. Step) were used as thresholds for clustering of the precomputed model

For the first step of CD-Hit, we considered the lowest % similarity that allows homogenous functional mapping using the ground truth information we had for evaluation, as described in the methods section. For the second step, which used a different sub-procedure (CD-Hit-2D), a similarity threshold of 65% was too low with the same parameters, and failed running for some of the proteins in the complete RefSeq database, therefore, a close value of 70% was chosen.

• Why are there 2 steps, with first some 43K proteins (how are they exactly selected? From NCBI’s Prokaryotic Reference Genomes?) and then with 1.7M?

Thank you for pointing this out. The first step uses the RefSeq non-redundant prokaryotic protein database (<https://www.ncbi.nlm.nih.gov/refseq/about/nonredundantproteins/> ) which is used to establish the clusters, whereas in the second step, all RefSeq proteins are clustered against this database using the selected representatives, yielding 1.7M proteins that are clustered overall. The first step allowed unique, non-redundant clusters, whereas the second step allowed expansion of these clusters and greater protein diversity within each cluster, for training of the k-mer frequency model. This is now described in the revised methods section:

“We first clustered 43,176 proteins from the NCBI RefSeq non-redundant microbial protein database^40^ using CD-HIT with a similarity threshold of 65% and a word size of 5. This first clustering step resulted in 32,308 clusters, allowing non-redundant clusters. In the second step, to further expand and diversify protein members within these clusters, we applied CD-HIT-2D^44^ with a 70% sequence similarity threshold to cluster all RefSeq microbial proteins against the previously selected cluster representatives (N=1,797,426 protein that were clustered, from RefSeq dataset downloaded in May 2023). A set of expanded clusters was created from this process such that the final dataset clusters 1,793,361 proteins into 32,308 functional groups.”

Since the 2-step clustering is non-standard and differs from plain CD-HIT, it would be good to get a sense of clustering quality as measured by a clustering coefficient like the Silhouette or Dunn index or homogeneity/separation ratio.”

We now explain the motivation for the 2-step clustering, which is using different datasets. We demonstrate the cluster quality in Figure 2b and Figure 2c by comparing to clusters from established reference databases (albeit with lower coverage compared to the database used by our model) and by assessing the homogeneity of protein annotations within the clusters. Silhouette or Dunn index assume a distance metric and centroids, which we do not have for our proteins and clusters. In lieu of this, we elected to infer quality by protein naming consistency.

Was the hyperparameter (k) evaluation done according to best practices? I.e., was it conducted with proper dataset splits (inner validation sets for model/hyperparameter selection and “outer” final test set(s), which was entirely unused(!) during hyperparameter selection? How sensitive are the results to a change of k?

The datasets used for benchmarking were completely unseen for hyperparameter search. The hyperparameter search was using AA segments from the protein database, as now described in the revised methods section:

“The value of k was chosen by using a hyperparameter search, by simulating truncations of each protein in the database into 50AA overlapping segments. k values of 3,4,5,6, were examined, and k=5 was selected as it optimized the performance on truncated reference AA sequences. We first obtained overlapping 5-mers of each protein amino acid sequence.”

Limitations should be clearer. Avoiding assembly is obiouslybeneficial from a computational point of view, but lacks the uniqueness/predictive power stemming from assembled contigs.It would be desirable, if that could be quantified.

The selection of 43K reference proteins most likely contains multi-domain proteins. There are occasions when read assembly is important:
1. Multidomain Proteins & Functional Context:
o Many functional proteins, especially in signaling (e.g., two-component systems) and metabolism, derive their activity from domain interactions.
o If sequence reads only cover individual domains, assigning function may be incomplete or misleading (e.g., distinguishing a full enzymatic complex from fragments).
o Assembling longer contigs helps reconstruct the true architecture of multidomain proteins.

2. Pathway Reconstruction & Gene Clustering:
o Some pathways rely on synteny (gene order and co-localization), which is lost in fragmented reads.
o Functional units like operons in prokaryotes may be misclassified if only single reads are used.

3. Taxonomic and Functional Coupling:
o If a gene is part of a mobile genetic element (e.g., plasmid, transposon), assembly can clarify if it belongs to a specific species or is horizontally transferred.

Another limitation is that beyond the precomputed protein clusters, novel proteins in metagenomes will be ignored. While this is common to many methods, it should probably be mentioned.

We appreciate this comment and agree that these are important limitations, and therefore added a discussion of these to revised manuscript:

“Third, although we did find evidence supporting some remote homology detection, like any method that relies on sequence comparisons *k*Mermaid cannot classify truly novel or unseen proteins. Last, there are several biological limitations of inferring function from short reads that *k*Mermaid does not address, including misclassification of multi-domain proteins and operons. These limitations will remain for any metagenomic protein mapping tool designed for short reads and researchers wanting a more thorough view of the functional content of metagenomes should consider methods that include some degree of contig assembly prior to classification^40–42^.”

Furthermore, our expanded benchmarking in the revised Figures 3,4 better demonstrate the utility of the approach for diverse use cases vs limitations.

It would be very useful if the presented work could assess the amount of misclassification coming from unassembled reads (vs assembled reads).

We have clarified in the text as well as the GitHub repo documentation that *k*Mermaid is designed for read mapping and application to assembled reads is out of the scope for this study. Contig mapping requires gene prediction before applying *k*Mermaid, and we have not tested this functionality in the current study.

Assessing the quality of the initial clustering is also important:

We demonstrate the cluster quality in Figure 2b and Figure 2c by comparing to clusters from established reference databases (albeit with lower coverage than our model) and by assessing the homogeneity of protein annotations within the clusters. Silhouette or Dunn index assume a distance metric and centroids, which we do not have for proteins and protein clusters. In lieu of this, we elected to infer quality by protein name search, etc.

Regarding memory consumption, DIAMONDs strategy seems a realistic approach (scaling with input, capping at 16GB). There should be a stronger motivation for a limitation to 2GB, an amount that is exceeded by nearly any reasonable machine in use these days. It is not clear how the 2GB memory requirement is achieved – streaming/generators?

While 16GB of RAM may be realistic for HPC users with more massive workstations, a total 16GB of RAM is currently a common configuration for many laptops (and exceeds the capacity of many older laptops), and thus is not realistic most for users that prefer a local analysis.

A central point is that existing methods (particularly BLASTX, not so sure about the other abovementioned methods) have a high percentage of ambiguous mappings (Fig. 2). Have the authors

We are unable to fully address this comment because it is incomplete, but we have clarified in the text what is meant by ambiguous mappings/multimaps, i.e. homologous proteins originating in different taxa but often having the same functional annotation (name) and showed the trend for DIAMOND in addition to BLASTX (Figure 2a).

Regarding speed comparisons, indexing is an extremely common method in databases to perform fast lookup. It is also used in [1]. kMermaid does not use indexing or other fast information retrieval methods hashing/LSH, sorting etc. It would be helpful to provide insides as to why such common/best practices were not deployed/necessary.

We appreciate this comment. This point was unclear due to incomplete description of the implementation of *k*Mermaid in the previous version of our manuscript. *k*Mermaid uses a nested hashing approach, which is now described in the revised methods section:

“The underlying model is then stored in a two-level hash map where the top-level map stores for each *k*-mer *k,* a map of clusters containing *k*, and the second level maps *k* and a clusters C containing *k*, to a cluster frequency *Ck*. The resulting nested hash map can be written as $Model\left[ k \right]\left[ C \right]\leftarrow{frequency}_{Ck}$.”

As well as in the pseudo-code provided in the supplementary information

There are a few technical issues with the installation. Both on Ubuntu 20.04 (as recommended) and Mac OS 15.3, large file installation did not seem to work as described in the installation instructions: even with git-lfs installed, the file kmermaid/db/kmer_model.pkl is only 134bytes and thus not useful (not readable with pickle). The alternative method using the wget command (as per the github install instructions) does not give a larger file.

Thank you for these comments and we apologize for the difficulty installing the tool. We have moved the corrupted file to Zenodo and improved the installation instructions on the GitHub readme.

[1] Steinegger, M., Söding, J. MMseqs2 enables sensitive protein sequence searching for the analysis of massive data sets. Nat Biotechnol 35, 1026–1028 (2017). <https://doi.org/10.1038/nbt.3988>
