# Supplementary material for: kMermaid: Ultrafast metagenomic read assignment to protein clusters by hashing of amino acid k-mer frequencies
Source: PLoS Comput Biol. 2025 Sep 11;21(9):e1013470. doi: 10.1371/journal.pcbi.1013470 (PMC12507277; doi:10.1371/journal.pcbi.1013470)
Supplement: Attachment — Submitted filename: response_to_reviewers_plos_comp_bio.docx [file pcbi.1013470.s010.docx]

We thank the reviewers for their additional comments and suggestions. We have addressed all points in this revision, which further clarifies the approach and supports the utility of *k*Mermaid. A detailed point-by-point response is provided below (responses in red).

**Reviewers' comments:**

Reviewer's Responses to Questions

Reviewer #1: I appreciate the authors’ considerable effort in improving the benchmark and the figures, and I think the authors have resolved most of my concerns.

Regarding my previous comments:

1. I agree with the authors that there is value in using the new database where proteins are clustered using AA words.

2. Thank you for your detailed clarification for the content in figure 2. There are some small typos: In Figure 2.a, “≤5” should be “≥5”, and in Figure 2.c, consider limiting the x-axis to be between 0 and 100%.

Thank you for pointing these out. We fixed the label for Figure 2a. Restricting the axis on Figure 3c would make it difficult to see the peak at 100%.

3. The benchmark in Figure 3 is clearer and shows that kMermaid is among the best performing tools in terms of sensitivity and coverage in most cases. The authors should specify how “% match” is calculated. Is it the same for all tools? If “% match” is the sensitivity at the cluster level for kMermaid, but at the protein level for the other tools, this might not be a fair comparison.

We appreciate this comment. Figure 3 compares the methods when employing those to the input and using the same reference database. While *k*Mermaid only maps reads to clusters, the other methods only map reads to proteins. We can only evaluate sensitivity in terms of the output given by the methods, which means sensitivity in cluster assignment for *k*Mermaid and in protein assignment for other methods, which correctly represent the true positive rate of the output of each of these methods. The improvement in sensitivity by cluster assignment is part of the problem *k*Mermaid is addressing. As shown in Figure 2a, the multimapping by other methods result with lower sensitivity, because only one origin of each read is a true positive. In the revised manuscript we added text to clarify this point:

“Thus, by resolving multi-mapped reads at the cluster level, *k*Mermaid shows improved sensitivity compared to methods that assign reads only to individual proteins”

Some additional typos and minor issues that I spotted:

1. In the “Creating kMermaid’s k-mer frequency cluster model using nested hashing” section, the authors used “for each k-mer k, a map of clusters containing k”, which is confusing as k can both indicate a k-mer and the length of a k-mer. Consider changing it to “for each k-mer w”.

Thank you for this suggestion. We agree that using different variables would improve clarity and have changed the text in the methods section and pseudocode in the Supplementary, such that w represents an individual k-mer and k represents the length of the k-mer.

2. In the “Assigning protein maps to reads using the pre-computed k-mer model” section, the author claimed that the score would be the sum of k-mer frequencies for the k-mers in the query sequence, but in the formula, the frequency is summed over $k_i\in C$ (all the k-mers in the cluster C), which is inconsistent.

Thank you for this comment. We edited this section in the revised manuscript, which is now better defined to clearly demonstrate that the frequency is summed over w in the query sequence

“To assign a read into a protein cluster, a six-frame translation is applied to a query sequence $r$ and translations containing a stop codon (truncated frames) are discarded. Next, all overlapping AA 5-mers are extracted from the non-truncated frames. A score representing the strength of a match between $r$ and each cluster $C$ is then calculated by the summation of the precomputed model *k*-mer frequencies for each *k*-mer $w$in the query sequence. The score $s_{r,C}$ for query sequence $r$and each cluster *C* is computed as:

$$s_{r,C}= \sum_{w\in r} {frequency}_{Cw}$$

where ${frequency}_{Cw}$ is the model frequency of each *k*-mer *w* from $r$ in cluster *C,* i.e. the average times *w* appears in proteins of *C*. Finally, the sequence is then assigned to the cluster with the global maximum score across all clusters (Figure 1; Supplementary information, Algorithm 2). *k*Mermaid annotates the query sequence by the cluster representative for the cluster corresponding to this maximum assignment score.“

3. Do the “composite score” and the “confidence score” refer to the same thing?

We previously used the term “composite score” to refer the assignment score of a query read to any cluster in the model, and “confidence score” to refer to the maximum composite score (i.e. confidence in the final assignment). We agree this could be somewhat confusing and have changed references to *k*Mermaid’s scoring singularly as “assignment score” throughout the text.

4. I’m still a bit confused by the description of hyperparameter search: “k=5 was selected as it optimized the performance on truncated reference AA sequences”. It might be better to specify which metric they are optimizing: is it based on cluster purity, or AUROC of the downstream classification task, or something else?

Thank you for this comment. The metric evaluated is accuracy in classifying truncated protein sequences, as most reads would likely be translated into such sub-sequences or truncations of proteins. We revised the respective methods section to clarify this point, and provide more details for the analysis underlying the selection of k:

“The value of k was chosen by using a hyperparameter search, by simulating truncations of each protein in the database into 50 overlapping AA segments. We evaluated k values of 3,4,5 and 6 for classifying truncated protein sequences, measuring accuracy as the fraction of truncated sequences which were correctly assigned to their cluster. Both k = 5 and k = 6 achieved similarly high accuracy (>0.99), but the number of k-mers increased sharply from 2,574,615 for k = 5 to 12,043,100 for k = 6. Therefore, k = 5 was selected, achieving high accuracy with substantially fewer parameters.”

5. “The cluster frequency” is better written as “The frequency of k-mers in the cluster”.

Thank you - we changed this section to be more explicit. The revised manuscript now states:

“The underlying model is then stored in a two-level hash map where the top-level map stores for each k-mer w, a map of clusters containing w, and the second level maps the clusters C containing w, to the frequency of w in C, Cw”

6. “The k-mer frequencies for each cluster C were defined by the count of the k-mer in the cluster C divided by the total number of proteins in the cluster.” Can this k-mer frequency be >1 if this k-mer appear multiple times in the same protein?

That is correct. We have added a note about this to the methods for clarity.

7. In Figure 3.c, consider using log scale for the y-axis.

Thank you for this suggestion. We have changed the y-axis scaling for Figure 3c.

Reviewer #2: This version addresses my previous concerns.

Reviewer #3: Most of the previous issues have been fully addressed. One remaining issue is the argument why Silhouette and Dunn index have not been calculate (the authors claim that it is impossible in the absence of centroids). No, it is not true that the Silhouette or Dunn index require the presence or calculation of centroids.

We agree with the referee that the Silhouette and Dunn indices do not require centroids and rely solely on a distance metric. We have now carefully evaluated this approach for our protein clustering analysis.

To compute pairwise distances between proteins, we selected the Jensen-Shannon distance (JSD) of 5-mer frequencies as the distance metric. Unlike set-based metrics like Jaccard distance, JSD better accounts for (1) k-mers appearing multiple times within a protein, capturing frequency differences, and (2) variations in protein length. For each protein, we constructed a feature vector of length 2,574,615 (the number of 5-mers in *k*Mermaid’s model), representing the frequency of each 5-mer in the sequence.

Using pairwise distances, a key challenge is the computational complexity of these analyses: The computational complexity of pairwise distance calculations is (p times N^2), where N is the number of proteins (1.8 million in our dataset) and p is the time to compute one JSD (0.01–0.1 seconds). For all pairwise comparisons 1.8M^2, this would require approximately 16.2–162 billion seconds (187,500–1,875,000 days) on a single CPU, or 1,870–10,875 days with 100-fold parallelization on our HPC. This is computationally infeasible.

To address this, we initially considered evaluating JSD between each of the 1.8M proteins and the 32K cluster representatives (treating representatives as proxies for centroids) to assess correct cluster assignments. These 1.8MX32K comparisons would require approximately 576 million–5.76 billion seconds sequentially, or 67–667 days with 100-fold parallelization, still exceeding practical limits.

We therefore adopted a subsampling strategy, randomly selecting 6,400 cluster representatives (~20% of 32,000 clusters). For each representative, we computed JSD between it and up to 19 of its cluster members (a cutoff chosen for computational feasibility) to obtain the average within-cluster distance. We then computed between-cluster distances to the other 6,399 representatives. For each cluster, the minimum mean between-cluster distance was used to compute the silhouette value. The mean silhouette value across all 6,400 clusters was 0.57 (SD = 0.13), indicating well-formed clusters. We visualized the silhouette distributions for “complete” clusters (≤19 members, i.e. within cluster JSD computed fully) and “subsampled” clusters (≥20 members, with up to 19 members sampled). Clusters of size 1 were excluded from visualization, as their silhouette values are uninformative, but included in minimum mean distance calculations to reduce bias.

The similarity between the distributions of complete and subsampled clusters suggests that our subsampling approach provides a reasonable estimate of the full dataset’s clustering quality. All silhouette values were positive, confirming that subsampled proteins were more similar to their own cluster representative than to others, based on 5-mer JSD. This supports the robustness of our clustering.

Due to the specified computational constraints, a comprehensive analysis on the full dataset was not feasible. These findings are noted here to provide further support for our clustering approach but are excluded from the main results to ensure focus on fully validated outcomes.
